# Delayed ocular disengagement from arousing scenes

**DOI:** 10.3389/fpsyg.2023.1297192

**Published:** 2023-12-21

**Authors:** Andrea De Cesarei, Nicola Sambuco, Stefania D’Ascenzo, Roberto Nicoletti, Maurizio Codispoti

**Affiliations:** ^1^Department of Psychology, University of Bologna, Bologna, Italy; ^2^Department of Philosophy and Communication Studies, University of Bologna, Bologna, Italy

**Keywords:** emotion, eye movements, arousal, negative bias, disengagement

## Abstract

Visual exploration of the world is supported by eye movements which can be speeded up or delayed depending on bottom-up stimulation, top-down goals, and prior associations. Previous studies observed faster initiation of saccades toward emotional than neutral natural scenes; however, less is known concerning saccades which originate from emotional, compared with neutral, scenes. Here, we addressed this issue by examining a task in which participants continuously moved their gaze from and toward pictures (natural scenes), which could be emotional or neutral, and changed position in every trial. Saccades were initiated later when the starting picture was emotional compared to neutral, and this slowing was associated with the arousal value of the picture, suggesting that ocular disengagement does not vary with stimulus valence but is affected by engaging picture contents such as erotica and threat/injuries.

## 1 Introduction

Efficient interaction with the environment requires the ability to engage, shift and disengage attention between elements in the visual field depending on their physical properties, goal-related pertinence, and history of selections (Awh et al., 2012). Attentional engagement, disengagement, and shift may involve overt movements of the eyes, head, and body (Yarbus, 1967; Belopolsky, 2015), in order to adapt to the temporal sequence of events, and can be modulated by bottom-up and top-down factors. It was observed that voluntary saccades toward emotionally arousing (pleasant and unpleasant) scenes presented in the periphery are initiated faster, compared with saccades toward neutral scenes (Nummenmaa et al., 2006; Calvo et al., 2008) when saccades start from a neutral object (e.g., a fixation cross). Consistently, it has been shown that the emotional significance of a scene can modulate behavioral correlates of attentional allocation (Sokolov, 1963; Lang et al., 1997; Bradley, 2009), such as in paradigms presenting emotional stimuli as distractors (De Cesarei and Codispoti, 2008; Codispoti et al., 2016; Micucci et al., 2020; Ferrari et al., 2022). Taken together, previous results indicate that the motivational relevance of a scene modulates attentional engagement.; When scanning the environment in our daily life, saccades often initiate from pleasant or unpleasant contents, yet results concerning ocular disengagement from emotional scenes are mixed. A previous study reported saccades originating from negative natural scenes being slower than saccades from positive pictures (Ferrari et al., 2016; for similar results in a study using schematic emotional faces see Belopolsky et al., 2011). However, two studies observed the opposite pattern, with slowest responses for neutral, compared with emotional, stimuli (Sears et al., 2010; West et al., 2011). Finally, no effect was observed in non-clinical participants in two studies examining ocular disengagement in depressed vs. control participants (Sanchez et al., 2013, 2017). Additionally, only a few studies used both pleasant, unpleasant, and neutral contents, thus allowing to disentangle an effect of arousal from an effect of valence, or emotional effects that are specific to events of negative valence (negative bias; Baumeister et al., 2001). Due to the variability in stimulus materials (e.g., faces vs. natural scenes) and experimental paradigms, and to the mixed pattern of findings, these previous results do not provide consistent evidence on the effects of emotion on ocular disengagement from natural scenes.; The present study examined the relationship between emotions and oculomotor behavior, asking participants to move their gaze between successively presented pictures, and systematically varying the emotional value (emotional vs. neutral) of pictures situated at the starting and at the ending point of the saccade. As we were interested in the disengagement from the current gazing location, we favored disengagement by prompting picture change with a gap, which acts as a warning signal that speeds up saccades (Kingstone and Klein, 1993). The study aimed to distinguish between effects that depend on a scene’s arousal value (i.e., similar for highly arousing pleasant and unpleasant scenes) from effects contingent to a specific valence (i.e., effects that are unique to negative contents). To this end, we presented pleasant and unpleasant natural scenes varying in emotional arousal (Bradley et al., 2001), together with neutral scenes. It may be expected that saccadic response times are slower when starting from pleasant and unpleasant, compared with neutral, contents if disengagement is modulated by arousal similar to attentional capture (arousal scenario; e.g., Lang et al., 1997). On the other hand, faster initiation of saccades is expected when starting from unpleasant compared with neutral and pleasant stimuli if ocular behavior reflects behavioral avoidance of threatening events or stimuli (valence scenario; e.g., Miller, 1956; Poncet et al., 2022).; In addition to the bottom-up modulation related with stimulus content, overt attention is top-down modulated by the expectation that a specific object will be presented at a spatial location, and this expectation is based on the previous learning; for instance, all stimuli that appear in a specific hemifield might share the same affective value (e.g., all pictures that appear on the left are pleasant), and this may prompt the top-down expectation that a stimulus appearing in the same hemifield will have the same affective value. Statistical learning of spatial contingencies has been well documented, both concerning the modulation of manual response times and of ocular overt behavior (Xu et al., 2023). More specifically, when a location in the visual field was spatially biased, it was observed that the deployment of attention to predictable locations took place faster than to unexpected locations (e.g., Pfeuffer et al., 2020; Boettcher et al., 2022; Xu et al., 2023). Here, we will examine the extent to which top-down (learned) contingencies between a spatial location and an emotional content add to the bottom-up effect of picture emotionality. To this end, for one group of participants (hemifield condition) we created an association between emotional content and spatial locations, while for another group of participants (random condition) each content could appear at any spatial location. If top-down statistical learning of spatial contingencies and bottom-up emotional modulation of ocular behavior are related, then we might expect more pronounced modulations of saccadic responses in the hemifield condition, in which emotional content and spatial position are associated.

## 2 Method

**Figure 1:**
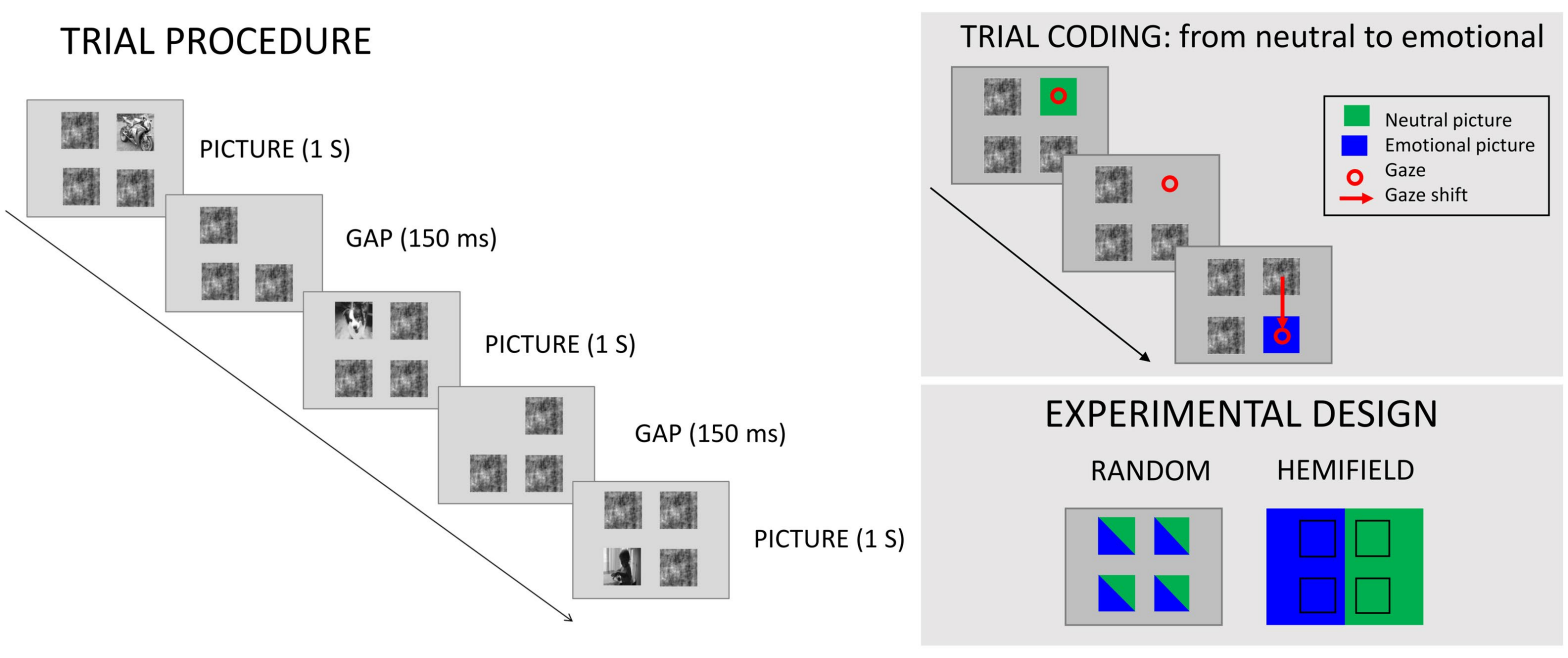
Procedure and design details. In each trial, a pattern of four images was presented, three of which consisted in a phase-scrambled image and one in a greyscale picture. Participants had to gaze at the picture as soon as it appeared; after a 1 s exposure time, the picture disappeared and was replaced by a 150 ms gap. After this short gap, the next pattern was presented, and participants had to gaze at the newly appeared picture. Trials were coded according to the valence of the starting and the ending point of the saccade; in the example on the upper right, the trial was coded as going from neutral to emotional. In the experimental design each of the four positions could either represent a neutral or an emotional image (random condition) or be associated with exclusively one emotional valence (hemifield condition).

A total of 59 participants took part in the study. Four participants were excluded because they did not complete the study or had less than 50% valid trials, leaving a total of 55 participants (*F* = 34; mean age = 22.3, SD = 2.5). Participants were randomly assigned to the hemifield or to the random group (*N*s = 26 and 29 respectively). Sample size was determined based on an *a-priori* power analysis (G*Power; Faul et al., 2007) on independent data with alpha = 0.05, power = 0.90, *η*^2^*_p_* = 0.05 (medium effect according to Cohen, 1969), and correlation among repeated measures = 0.8, for a repeated-measures ANOVA with a two-levels between-participants factor and within-participants numerator degrees of freedom = 1. This analysis indicated a minimum of 24 participants, consistently with previous studies which examined the emotional modulation of eye movements (Calvo et al., 2008; Bradley et al., 2011). All participants had normal or corrected-to-normal vision, and none of them reported current or past neurological or psychopathological problems. Data and study materials are available upon request to the corresponding author. The experimental protocol was approved by the Bioethical Committee of the University of Bologna.; A total of 1,024 pictures were selected from the Internet and from existing databases of emotional stimuli (IAPS, international affective picture system; Lang et al., 2008), comprising pleasant (*N* = 256), neutral (*N* = 512), and unpleasant (*N* = 256) pictures. Picture count for each category was: erotica, *N* = 84; romance, *N* = 86; babies, *N* = 86; neutral pictures, further divided in moving people, *N* = 169; static people indoors, *N* = 173 pictures; static people outdoors, *N* = 170; injuries, *N* = 84; illness, *N* = 86; human attack, *N* = 86. All pictures were converted to grayscale and equated in brightness and contrast (pixel intensity *M* = 127.5, SD = 4.51).; In each trial, a pattern consisting of four images was presented to participants. These images were positioned at the edges of an imaginary square. Out of these four images, one represented a real-world natural scene, while the other three represented a phase-scrambled image. The task of participants was to gaze at the natural image as soon as it appeared. The image pattern was displayed for 1 s, and a 150 ms gap was presented between two successive trials. During the gap, only the previous picture would disappear, while the three phase-scrambled patches remained onscreen. When the next image array (three scrambled patches plus a picture in a different position) appeared, participants had to gaze at the newly appeared picture. It was made explicit to participants that two subsequent image positions were never at diagonally opposite edges of the imaginary square, as this would have implied a longer trajectory to be made compared with horizontal or vertical eye movements (Figure 1). Horizontal and vertical movements were balanced within each condition. Picture size was 9.9° by 9.9°, and distance between the inner edges of pictures was 5°. Each picture was presented only once to each participant. Before starting the experiment, a practice phase was performed, showing a total of 20 additional neutral pictures which were not repeated during the experiment.

A total of two experimental blocks were performed, each comprising 512 trials. These blocks differed in the valence of the images presented: pleasant and neutral pictures were presented in one block, and unpleasant and neutral pictures in the other, with the order of blocks balanced across participants. Participants were assigned to one of two experimental groups, so that for one group (random condition) each spatial position could portray any picture valence, while for the other group (hemifield condition) each specific spatial position would only be associated with an emotional valence (e.g., pleasant for left). For participants in the hemifield condition, all hemifields were assigned to each picture valence in within-participant sub-blocks.; Eye movements were recorded at 250 Hz sampling rate from a SMI RED 500 eye tracking system positioned 60 cm away from the participant, below a 22-in LCD monitor. The first experimental block was preceded by a four-point calibration procedure. Average drift during the calibration was 0.51° (SD = 0.26°; x: *M* = 0.54°, SD = 0.24°; y: *M* = 0.48°, SD = 0.28°). The latency of initiation of each saccade was analyzed using ILAB (Gitelman, 2002). In order to focus on accurate trials in which saccadic response time was unconfounded by an erroneous picture position or an erroneous ending position, only saccades that started and ended in the correct positions were analyzed. Saccades which were faster than 80 ms from the onset of the target array or slower than 2.5 SD from the participant average were excluded. Overall, 22.7% of the total trials were rejected (random condition = 24.7%, hemifield condition = 22.38%), the main reason for rejection being not starting or ending in the desired position (*M* = 22.96%), followed by extremely fast or slow responses (*M* = 2.6%). An ANOVA with Huynh–Feldt correction was conducted with the within-participant factors Block Valence (whether emotional pictures in the block portrayed pleasant or unpleasant contents), Starting Picture Arousal (arousal of the starting picture: arousing vs. neutral), and Ending Picture Arousal (arousal of the landing picture: arousing vs. neutral), and the between-subjects factor Presentation Type (random vs. hemifield). Partial eta squared (*η*^2^*_p_*) is reported as a measure of effect size.

## 3 Results

**Figure 2:**
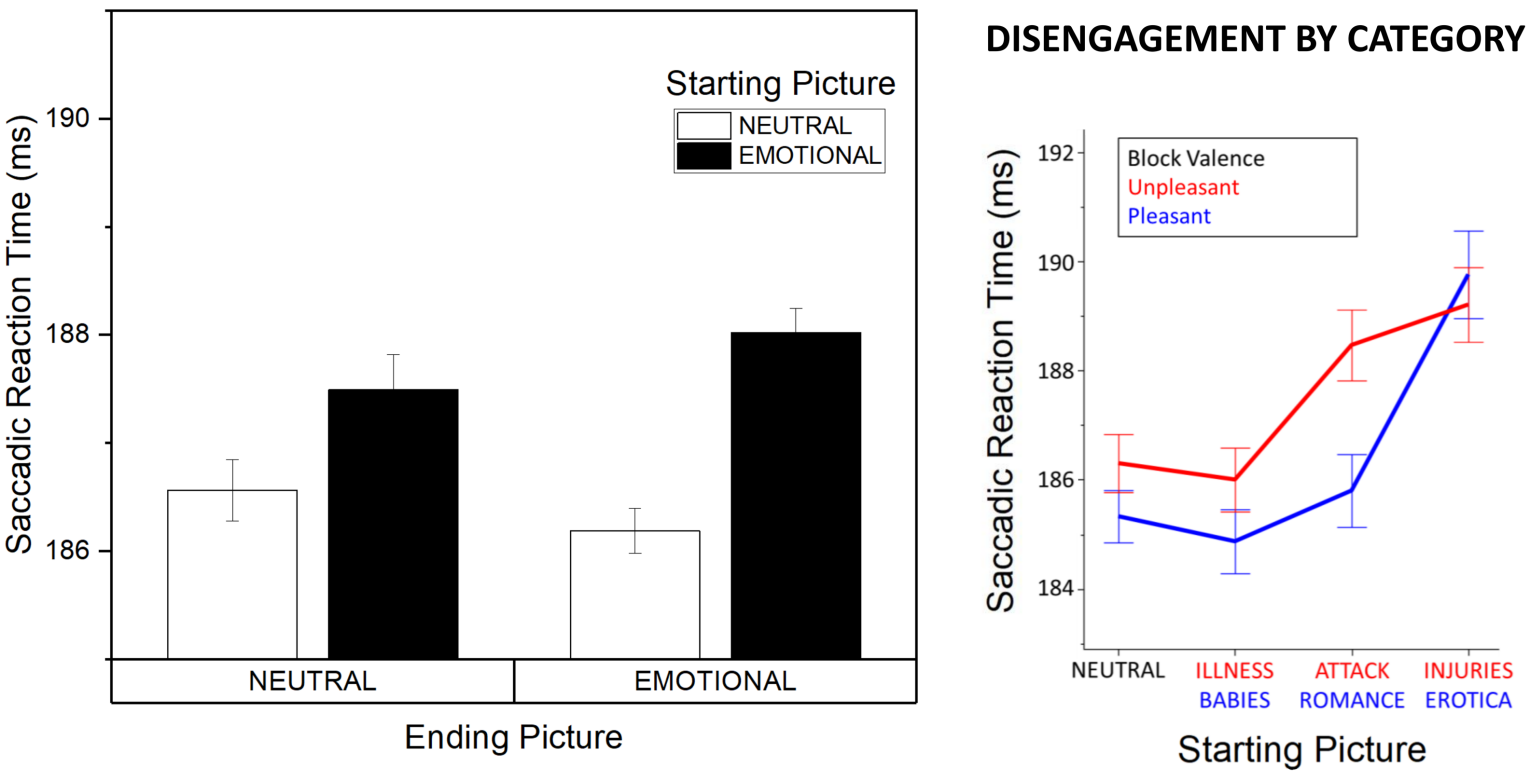
Affective modulation of saccadic latency. Left: effects of emotionality (emotional vs. neutral) of the starting and landing picture on saccadic latency. Right: effects of affective category on ocular disengagement. Error bars represent within-participant standard errors (Cousineau, 2005).

Significantly slower initiation of saccades was observed when starting from emotional (*M* = 187.06, SD = 12.90), compared with neutral (*M* = 185.61, SD = 12.53) pictures, Starting Picture Arousal, *F*(1, 53) = 25.62, *p* < 0.001, *η*^2^*_p_* = 0.326. This effect was not modulated by Block Valence, *F*(1, 53) = 0.053, *p* = 0.819, *η*^2^*_p_* = 0.001, and no interaction of Presentation Type and Starting Picture Arousal was observed, *F*(1, 53) = 0.665, *p* = 0.419, *η*^2^*_p_* = 0.012. Consistently, a significant effect of Starting Picture Arousal was observed both in the random and in the hemifield condition, *F*(1, 28) = 10.31, *p* = 0.003, *η*^2^*_p_* = 0.269 and *F*(1, 25) = 15.11, p = 0.001, *η*^2^*_p_* = 0.377 respectively, and for pleasant and unpleasant blocks, *F*(1, 53) = 13.98, p < 0.001, *η*^2^*_p_* = 0.209 and *F*(1, 53) = 14.12, p < 0.001, *η*^2^*_p_* = 0.21, respectively. No effects of Ending Picture Arousal were observed, *F*(1, 53) = 0.019, *p* = 0.892, *η*^2^*_p_* < 0.001. A significant interaction between Block Valence and Presentation Type was observed, *F*(1, 53) = 5.75, *p* = 0.020, *η*^2^*_p_* = 0.098. Following on this significant interaction, we observed a significant Block Valence effect in the random condition, *F*(1, 28) = 4.91, *p* = 0.035, *η*^2^*_p_* = 0.149, with slower initiation of saccades in the block comprising unpleasant and neutral (*M* = 190.47, SD = 13.52), compared with the block comprising pleasant and neutral (*M* = 187.54, SD = 12.21), pictures. No effect of Block Valence was observed in the hemifield condition, *F*(1, 25) = 1.26, *p* = 0.272, *η*^2^*_p_* = 0.048.; As a supplementary control analysis, we aimed to substantiate the effect of Starting Picture Arousal against some of the possible confounding perceptual factors, i.e., complexity and composition. Complexity was measured as contrast energy and spatial coherence (Groen et al., 2012), while composition was measured as spectral power in three spatial frequency bands (low: 2.94–5.36 log_2_ cpi; medium: 5.36–7.6 log_2_ cpi; high: 7.6–9.96 log_2_ cpi; De Cesarei and Loftus, 2011). A linear regression at the single image level was run with factors Valence (three levels: pleasant, neutral, unpleasant, dummy coded in respect to neutrals), spatial coherence, contrast energy and power in the three frequency bands. A significant effect of Valence was observed, standardized beta = −0.112, *p* < 0.001, but not for all perceptual control variables, standardized betas < −0.056, *p*s > 0.230.; To further characterize the observed effect of Starting Picture Arousal, follow-up analyses were conducted to assess the category-specific differences in determining the initiation of saccadic responses (Figure 2). Specifically, the category of the starting picture was coded based on previous studies (Bradley et al., 2001; Schupp et al., 2004; Codispoti and De Cesarei, 2007; De Cesarei and Codispoti, 2011): (1) people in everyday activities (neutral); (2) babies and illness (low arousing), (3) romance and attack (mid arousing), (4) erotica and injuries (highly arousing). An ANOVA with factors Starting Picture Category (4 levels), Block Valence (pleasant vs. unpleasant), and Presentation Type (random vs. hemifield) was conducted, revealing a main effect of Starting Picture Category, *F*(3, 159) = 25.71, *p* < 0.001, *η*^2^*_p_* = 0.323, with slower saccade initiation when starting from mid (romance, attack) or highly arousing contents (erotica, injuries) compared with low arousing (babies, illness) or neutral contents (all *p*s < 0.004); slower saccadic initiation was found for erotica and injuries compared with all other categories, *p*s < 0.001, and no significant difference between neutral and low arousing contents was observed, *p* = 0.393. A significant interaction between Block Valence and Starting Picture Category was observed, *F*(3, 159) = 3.31, *p* = 0.030, *η*^2^*_p_* = 0.059, followed by significant effects of Starting Picture Content in both the pleasant and unpleasant block, *F*s(3, 159) > 10.35, *p*s < 0.001, *η*^2^*_p_*s > 0.163. Follow-up analyses revealed that saccadic initiation in the unpleasant block was slowest for injuries and attack compared with neutral and illness, *p*s < 0.001, while no significant difference was observed between pictures of injuries and attack, *p* = 0.387 and between neutral scenes and pictures of illness, *p* = 0.594. In the pleasant block, slower saccadic initiation was observed for erotica compared to all other contents (romance, babies, and neutral; all *p*s < 0.001), and no difference among other contents, *p*s > 0.322. Finally, a linear contrast analysis was conducted (neutral < low < mid < highly arousing), which indicated a significant linear effect of Starting Picture Content, *F*(1, 53) = 60.20, *p* < 0.001, *η*^2^*_p_* = 0.532, that did not interact with Block Valence, linear x linear contrast *F*(1, 53) = 0.65, *p* = 0.424, *η*^2^*_p_* = 0.012, and was followed by significant linear contrasts in both the pleasant and in the unpleasant block, Starting Picture Content *F*(1, 53) = 30.33, *p* < 0.001, *η*^2^*_p_* = 0.364 and *F*(1, 53) = 20.89, *p* < 0.001, *η*^2^*_p_* = 0.283, respectively.

## 4 Discussion

The present study sought to investigate affective modulation of ocular engagement/ disengagement. Using natural scenes, we observed that the arousing value of the starting image slowed down ocular disengagement, and this result was replicated for both positive and negative contexts, and for random and hemifield presentation type. On the other hand, no modulation of ocular engagement was observed in these data. Systematically associating a picture location to a picture content did not determine stronger emotion effects on ocular engagement or disengagement.; The observed slowing of disengagement for emotional compared with neutral pictures is consistent with observations in the field of motivated attention and suggests that high- arousal content can hold attention more than low arousing content (Lang et al., 1997; Bradley, 2009). Consistently, longer viewing time has been reported for emotional compared with neutral scenes (Lang et al., 1993). Concerning saccadic response times however, previous studies yielded an inconsistent pattern of results (Belopolsky et al., 2011; West et al., 2011; Machado-Pinheiro et al., 2013; Sanchez et al., 2013, 2017; Ferrari et al., 2016; Sanchez-Lopez et al., 2019). Here, in all experimental conditions slower disengagement was observed for emotional compared with neutral stimuli, and within each emotional valence, a linear slowing of saccadic latencies was observed as arousal increased.; The affective modulation of disengagement was replicated in pleasant and unpleasant blocks, and we also observed that associating a spatial position with an affective content (e.g., unpleasant pictures appearing always on the right) did not modulate the magnitude or direction of emotional effects. These results suggest that, while ocular disengagement can be modulated by picture arousal, its latency does not depend on picture valence. Henceforth, the present data do not support the possibility of a specific effect of unpleasant emotions on ocular disengagement (negative bias, Baumeister et al., 2001; Poncet et al., 2022), and rather suggest that arousal modulates ocular disengagement. However, consistently with previous reports (Schupp et al., 2004), we observed that while slower disengagement for appetitive contents was specific to the highest arousing category (i.e., erotica), effects on the defensive side were observed for highly and mid-arousing pictures (i.e., pictures of injury and overt attack). This pattern of results might indicate that, while modulation of disengagement is not specific to negative contents, the attentional call from aversive contents is broader, i.e., generalizes to an higher number of categories, than that from appetitive ones (Schupp et al., 2004).; Here, we observed slower disengagement from several contents (compared with neutrals), both on the pleasant and unpleasant side of the valence continuum: attack, injuries, erotica. Notably, these natural scenes vary considerably compared with each other in terms of perceptual composition and structure; however, despite these intra-category differences natural scenes still retain statistical regularities that allow one to distinguish between them (scene statistics; e.g., De Cesarei et al., 2017), and may contain exogenous attentional cues. However, pictures in the present study were devoid of color-diagnostic information, and brightness and contrast were adjusted to the same values, therefore limiting the bottom-up effects of these variables. Also, a regression analysis failed to show any effect of picture complexity (contrast energy and spatial coherence; Groen et al., 2012) or power in three spatial frequency bands (De Cesarei et al., 2017) that might explain ocular disengagement. Finally, the possibility that scene statistics or exogenous cues modulate exploration and response concern the initial time period once the scene has appeared (attentional engagement; scene exploration), while the effects that are reported here are related to disengagement, which is more sensitive to top-down than to bottom-up factors (Brockmole and Boot, 2009). Taking these results together, the disengagement modulation which is observed here is more consistent with previous studies in the field of motivated attention than with a perceptual account (Lang et al., 1997; Bradley, 2009).; In the present study, the temporal modulation of saccadic latency is of limited magnitude and in the direction of slower saccade initiation when starting from emotional compared with neutral scenes. In terms of consistency, emotional modulation of ocular disengagement was replicated in the random and hemifield conditions, and for pleasant and unpleasant stimuli. On the other hand, we did not observe a modulation of engagement nor a modulation by spatial contingencies (random vs. hemifield condition). In contrast, previous studies observed modulation of saccadic latencies by arousal (e.g., Nummenmaa et al., 2006; Calvo et al., 2008), with faster saccadic latencies to emotional compared with neutral stimuli, as well as facilitation (speeding up) of saccade initiation when spatial location was predictable (Pfeuffer et al., 2020; Boettcher et al., 2022). One explanation for the lack of facilitation in the present study as well as for the limited magnitude of saccadic modulation concerns the dynamic paradigm employed in the present study; specifically, the use of a pre-stimulus gap and the regular pacing of stimulus onset/offset could have promoted disengagement and left limited room for a further facilitation by emotion. On the other hand, it might still be possible to slow down saccadic initiation when disengaging from an already decoded picture. Future studies might explore this issue by manipulating the use of a gap and by increasing the variability in the temporal pacing of the scenes.; In terms of constraints on generality (COG; Simons et al., 2017), here we used pictures of natural scenes from different affective categories, including highly arousing ones (erotica, injuries). Participants were an unselected sample from the University of Bologna, and other published studies on the same population indicated that these stimulus categories modulate affective states (e.g., Codispoti and De Cesarei, 2007). Highly arousing pictures elicited the most pronounced disengagement effect. We expect the results to generalize to situations in which participants view similarly arousing sets of stimuli.; Taken together, the present results show slower disengagement from emotional compared with neutral stimuli, and within each emotional valence (pleasant and unpleasant), a linear slowing of saccadic latencies as arousal increased across picture contents. Ocular disengagement seems not to vary with stimulus valence but is affected by engaging picture contents such as erotica and threat/injuries.

## Data availability statement

The raw data supporting the conclusions of this article will be made available by the authors, without undue reservation.

## Ethics statement

The studies involving humans were approved by Bioethics Committee of the University of Bologna. The studies were conducted in accordance with the local legislation and institutional requirements. The participants provided their written informed consent to participate in this study.

## Author contributions

ADC: Conceptualization, Data curation, Formal analysis, Funding acquisition, Investigation, Methodology, Project administration, Resources, Software, Supervision, Validation, Writing – original draft, Writing – review & editing, Visualization. NS: Data curation, Investigation, Methodology, Validation, Writing – review & editing, Visualization. SD’A: Investigation, Writing – review & editing. RN: Conceptualization, Resources, Writing – review & editing. MC: Conceptualization, Data curation, Formal Analysis, Funding acquisition, Investigation, Methodology, Project administration, Resources, Software, Supervision, Validation, Writing – original draft, Writing – review & editing, Visualization.
